# Bioeconomy-Based Approaches for the Microbial Valorization of Citrus Processing Waste

**DOI:** 10.3390/microorganisms13081891

**Published:** 2025-08-13

**Authors:** Ioannis Stavrakakis, Paraschos Melidis, Nektarios Kavroulakis, Michael Goliomytis, Panagiotis Simitzis, Spyridon Ntougias

**Affiliations:** 1 Laboratory of Wastewater Management and Treatment Technologies, Department of Environmental Engineering, Democritus University of Thrace, Vas. Sofias 12, 67132 Xanthi, Greece; istavrak@env.duth.gr (I.S.); pmelidis@env.duth.gr (P.M.); 2 Institute for Olive Tree, Subtropical Plants and Viticulture, Hellenic Agricultural Organization—Dimitra (ELGO-DIMITRA), Agrokipio-Souda, 73164 Chania, Greece; kavroulakis@elgo.gr; 3 Laboratory of Animal Breeding and Husbandry, Department of Animal Science, Agricultural University of Athens, 75 Iera Odos, 11855 Athens, Greece; mgolio@aua.gr (M.G.); pansimitzis@aua.gr (P.S.)

**Keywords:** citrus processing waste, solid-state fermentation, cellulose degradation, antioxidant activity, biomass valorization, bioeconomy concept

## Abstract

The citrus processing industry is an economically important agro-industrial sector worldwide; however, it produces significant amounts of waste annually. The biorefinery concept and the recovery of bio-based materials from agro-industrial residues, including citrus processing waste, are emphasized in the European Green Deal, reflecting the EU’s commitment to fostering circularity. Biotreatment of citrus processing waste, including bioconversion into biomethane, biohydrogen, bioethanol and biodiesel, has been applied to valorize biomass for energy recovery. It can also be composted into a valuable soil conditioners and fertilizers, while raw and fermented citrus residues may exhibit phytoprotective activity. Citrus-derived residues can be converted into materials such as nanoparticles with adsorptive capacity for heavy metals and recalcitrant organic pollutants, and materials with antimicrobial properties against various microbial pathogens, or the potential to remove antibiotic-resistance genes (ARGs) from wastewater. Indeed, citrus residues are an ideal source of industrial biomolecules, like pectin, and the recovery of bioactive compounds with added value in food processing industry. Citrus processing waste can also serve as a source for isolating specialized microbial starter cultures or as a substrate for the growth of bioplastic-producing microorganisms. Solid-state fermentation of citrus residues can enhance the production of hydrolytic enzymes, with applications in food and environmental technology, as well as in animal feed. Certain fermented products also exhibit antioxidant properties. Citrus processing waste may be used as alternative feedstuff that potentially improves the oxidative stability and quality of animal products.

## 1. Citrus Processing Waste

**Figure 1:**
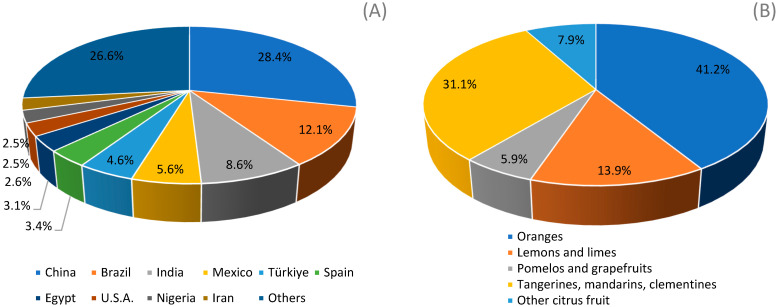
The major citrus producing countries (**A**) and citrus crops worldwide (**B**).

The citrus processing industry is an economically important sector worldwide and a key agro-industrial sector in the Mediterranean region. In particular, China, the Mediterranean countries, Brazil, India, the United States and Mexico produce more than 80% of the world’s citrus crops, with orange being the most abundant citrus fruit in the globe [1] (Figure 1).

As a consequence, a massive amount of waste is generated annually by the citrus processing industry, which is characterized by high content of pectin, cellulose and hemicellulose. The peel portion of various citrus fruits and the key bioactive compounds as well as pectin, lignocellulose and lipid/fat content of the peels of the major citrus crops are illustrated in Table 1.

For instance, orange peels contain a high pectin content, followed by hemicellulose and cellulose, each constituting nearly 10% of the composition, whereas soluble sugars represent up to 7% of the content [37]. Moreover, D- limonene and hesperidin are the key bioactive compounds found in the peels of the major citrus crops (Table 1).

Thus, more than 60 million tons of citrus waste are generated annually worldwide by this agro-industrial sector [49]. In Greece, more than one million tons of citrus fruits, with most of them being oranges, were produced in 2023 [1], of which approximately half were considered as food waste. Land disposal of untreated citrus processing waste may induce adverse effects on soil, compromising soil fertility and health [50]. Orange processing waste disposal can induce detrimental effects on soil fauna, e.g., on the earthworm *Lumbricus terrestris* [50]. Due to citrus processing waste decomposition, the rich in organic matter leachate generated by heavy rainfall can lead to increased total suspended solids and under certain circumstances, to oxygen depletion in local aquatic bodies [51]. Soil dumping of citrus processing waste can also result in pH decrease, affecting soil microbial community structure and nutrient cycling processes [51]. The presence of residual pesticides in citrus crops [52] may further affect soil fertility and quality if the waste is improperly disposed off. Thus, proper treatment of citrus processing waste is required to prevent soil deterioration and improve soil health for sustainable land use.

## 2. Legislative Framework for the Valorization of Citrus Processing Waste in the EU

The current EU strategy towards sustainable use of organic waste as input material, shifting from a linear to a circular economy and bioeconomy model in food waste management and valorization. Towards a circular economy—waste management of food waste in the EU, anaerobic digestion for energy and fuels is “near the bottom of the bio-refining value pyramid” [53], whereas the recovery of high value-added substrates and biological agents for enhanced plant productivity as well as for improved food and animal feed is positioned on the top of the pyramid. Thus, the vast streams of citrus processing wastes should be valorized within the circular economy prism.

The Circular Economy Action Plan [54], as part of the European Green Deal, prioritizes the valorization of bio-waste, with a particular focus on the food industry and food supply chain sectors. Biorefineries’ concept and recovery of bio-based materials from agro-industrial by-products and residues are prominently featured in European Green Deal, reflecting the EU’s commitment to fostering a circular bioeconomy. In this direction, the EU Bioeconomy Strategy encourages local bioeconomy development through the sustainable use of biological resources, unlocking the biotechnological potential of microbial inocula to produce innovative products and converting the wastes and by-products of the agri-food sector into high value-added products. In alignment with the implementation of Fertilizing Products Regulation (Regulation (EU) 2019/1009) [55] and Animal By-Products Regulation (Regulation (EC) No 1069/2009) [56], there is a growing need for efficient, cost-effective, green technologies capable of biotransforming citrus processing waste into high value-added products, serving as plant-protective agents in sustainable agriculture, as well as a safe and nutritionally valuable component in animal feed. Although EU policy through the Green Deal supports circular bioeconomy approaches in valorizing citrus processing waste, practical implementation of such methods faces barriers, like regulatory uncertainty, misaligned incentives and lengthy product safety approvals for food and feed, as well as for biobased materials, a fact that may increase time to market and overall costs, posing an obstacle for private investors [57].

## 3. Biomass Valorization of Citrus Processing Waste for Energy Recovery

Anaerobic digestion can be considered as an environmental-friendly bioprocessing approach for citrus processing waste [58,59]. Limonene, a major component of citrus essential oils, can pose an obstacle in the anaerobic digestion of such waste due to its inhibitory properties [60]. On the other hand, Jiménez-Castro et al. [61] carried out two-stage anaerobic digestion of orange residues without the need of pretreatment.

Dark fermentation for biohydrogen production is also an alternative treatment method for citrus processing waste [62]. Camargo et al. [63] reported on biohydrogen production from citrus peels through dark fermentation preliminarily carried out by *Clostridium* and *Paraclostridium* strains. However, pretreatment steps are often required to enhance biohydrogen production during dark fermentation of citrus processing waste [64]. Indeed, the high essential oil content of citrus processing waste can inhibit its bioconversion to biomethane or biohydrogen, often requiring pretreatment steps that raise operating cost.

Bioethanol is another valuable biomolecule produced during yeast fermentation of citrus processing waste for energy generation [65]. For instance, orange peels were served as immobilization carriers of *Saccharomyces* yeasts to act as biocatalysts in alcoholic fermentation [66]. However, the removal of D-limonene is still a necessary step to enhance bioethanol yields.

Citrus processing waste can serve as substrate for oleaginous yeasts cultivation for further use in biodiesel production [67]. Cultivation of *Candida parapsilosis* Y19 on orange peels resulted in increased lipid content and a fatty acid composition consisting of unsaturated fatty acids, mainly oleic acid, suggesting that orange processing residues can serve as suitable substrates for growth of oleaginous yeasts for biodiesel production. In particular, they served as growth substrate for enhanced lipid production during fermentation with the oleaginous yeast *C. parapsilosis*, reporting total lipids production of 4.78 g/L that corresponded to lipid content in the microbial biomass of 39% [68]. Seeds of *Citrus sinensis* were also found to be rich in lipids, consisting of 37% of their content, with the lipid fraction comprising linoleic, palmitic and oleic acids [69], making them suitable for biodiesel production.

Bioconversion of citrus processing waste through ensiling can resulting in bioethanol and lactic acid production [70]. In this case, volatile solids and limonene can be reduced by approximately 65% and 75%, respectively, so increasing the anaerobic digestibility of the silage [71]. The main bioenergy recovery approaches from citrus processing waste are presented in Table 2.

## 4. Bioconversion of Citrus Processing Waste for Enhanced Soil Fertility and Phytoprotective Properties

Biotreated citrus processing waste can enhance soil fertility and induce phytoprotection. It has been found that biotreated citrus waste can improve physical properties, like soil porosity and water retention, as well as nutrient availability, resulted in enhanced microbial activity and population of beneficial microorganisms [72]. Interestingly, Kato-Noguchi and Kato [73] attributed the phytoprotective properties of citrus processing waste against weeds, herbivore insects, parasitic nematodes and phytopathogenic fungi to the direct effects of some compounds of these residues, like essential oils.

Co-composting is an aerobic treatment method for citrus processing waste, in which citrus peels are initially colonized by mesophilic yeasts, followed by thermophilic microbiota, resulting in a mature compost with acceptable phytotoxicity levels [74]. Vermicomposting with the earthworm *Eisenia fetida* is also a composting approach applied for treating orange processing waste [75]. The application of compost derived from citrus processing waste, following pH adjustment with phosphoric acid, resulted in optimal seedling development in tomato and zucchini at a 7.5% compost-amended substrate [76]. By applying 4 kg of orange waste per square meter of land, Tuttobene et al. [77] found that durum wheat yields were comparable to those achieved with mineral fertilizer.

Citrus processing waste can also be applied in weed management strategies. Shehata et al. [78] found that orange processing waste can contribute to weed control during onion cultivation, reporting double bulb yield. Ugolini et al. [79] found that an orange juice processing residue rich in limonene could inhibit the germination of the weed *Chenopodium album* and delay the germination in *Lactuca sativa* in manner that its plant biomass was not affected, thus exhibiting bioherbicidal properties.

Agricultural solid wastes, including citrus processing residues, have shown potential for the adsorption and partial removal of pesticides such as diazinon and parathion. This suggests a possible low-cost, sustainable approach for mitigating pesticide contamination in water and soil systems [80]. Essential oils from citrus processing waste have the potential to be applied as bioinsecticides. For example, *Citrus aurantium* extract has been used for the phytoprotection of chickpea plants against *Callosobruchus maculatus* [81]. Essential oils from citrus peels are also capable of preventing post-harvest plant diseases, such as anthracnose induced by *Colletotrichum gloeosporioides* and *C. scovillei* [82]. Sala et al. [83] used agro-industrial by-products, including orange peels, as growth substrates for the cultivation of bioprotective fungal agents *Beauveria bassiana* and *Trichoderma harzianum*. Even though nutrient immobilization or phytotoxic effects induced by residual essential oils may occur during land application. The bioconversion of citrus processing waste to soil fertility enhancers and phytoprotective agents is illustrated in Table 3.

## 5. Valorization of Citrus Processing Waste into Biobased Polymers, Antimicrobial Materials, and Adsorbents

Citrus processing waste can be used in sustainable applications within the prism of circular economy [84]. Citrus peels can be used as substrate to enhance the growth of polyhydroxyalkanoate (PHA)-producing microbes for bioplastics production. For instance, a Bacillus cereus strain growing on orange peels produced more than 0.4 g/kg polyhydroxybutyrate (PHB) [85]. A biocomposite consisting of Arabic gum and carboxymethyl cellulose as well as orange peel extract exerted antimicrobial properties against *Salmonella enterica* and *Escherichia coli* O157 and high antioxidant activity (0.45 mM Trolox/mg extract), also having ameliorated barrier properties regarding water vapor and oxygen transmission rates [86]. Pagliarini et al. [87] fabricated a biobased polymer composite consisted of poly(butylene succinate-co-adipate) and up to 20% *w*/*w* orange peels to be used as natural filler, exhibiting both antioxidant and antibacterial activity. Citrus by-products are also considered as eco-friendly resources for producing functional and smart food packaging [88]. Citrus processing waste essential oils can also be used as antimicrobial agents in food-packaging applications. Interestingly, waste eggshell together with essential oil from orange peels, to act as a bioactive agent, and pectin, has been used to make a biocomposite film [89]. This fabricated film performed effectively under hydrological stress and exhibited antimicrobial properties against *Staphylococcus aureus* and *Bacillus cereus* [89]. Moreover, submerged fermentation of orange processing waste using *Bacillus haynesii* E1 resulted in the production of a biosurfactant compound [90].

Citrus-derived residues can be converted into material with antimicrobial properties against various microbial pathogens. Notably, orange peel extracts have been used for the synthesis of nanoparticles with antimicrobial properties. Kifle et al. [91] recently synthesized silver nanoparticles (AgNPs) using the extract derived from *Citrus sinensis* peels, exhibiting protecting properties against bacterial pathogens, i.e., *Bacillus cereus*, *Escherichia coli*, *Morganella morganii* and *Staphylococcus aureus*, and spoilage fungi like *Alternaria alternata*, *Aspergillus niger*, *Fusarium oxysporum* and *Penicillium digitatum*. Citrus-derived, chitosan-coated, selenium nanocomposite was fabricated and tested as a fungicide against common plant pathogens, such as the fungus *Sclerotinia sclerotiorum*, resulting in almost complete pathogen suppression at a minimum inhibitory concentration of 0.5 ppm [92].

Citrus processing waste can find applications as adsorbent material to remove heavy metals and recalcitrant organic pollutants. Activated carbon derived from orange peels and modified by TiO_2_ has been examined in terms of its ability to remove As from water. In particular, a maximum adsorbent capacity of 10.9 mg/g was recorded by applying an adsorbent concentration of 3.3 g/L in the presence of 50 mg As/L, under a treatment time of approximately 5 h and pH 4.2 [93]. Kukowska et al. [94] stated that activated carbon derived from orange peels through microwave furnace activation at 800 °C can serve as an efficient, environmental-friendly adsorbent, reporting arsenic (As) (V), selenium (Se) (IV), copper (Cu) (II) and cadmium (Cd) (II) removal efficiencies greater than 15%, 20%, 98% and 80% when metal concentration of 200 μg/L was treated with 0.02 g orange peel-derived activated carbon. Bouchelkia et al. [95] reported that orange peels can be used as bioadsorbents, resulting in removing approximately 112 mg methylene blue dye/g of adsorbent. Valorization of citrus processing residues can be achieved by converting into innovative nanoporous materials [96]. Orange peels have been utilized to fabricate organometallic adsorbent nanomaterials like ZnO-based orange peel-derived composites (ZnO-NR@PC) to serve as porous material of high adsorbent efficacy and stability, with removal efficiency greater than 90% in the case of the dyes crystal violet and methylene blue [97]. Moreover, biochar made from orange peels was capable of adsorbing extracellular DNA, indicating its potential to reduce the environmental dissemination of antibiotic-resistance genes (ARGs) [98].

Citrus processing residues can also be transformed into high-sensitivity sensors for monitoring pollution. An electrochemical nitrate sensor was developed by using activated carbon derived from orange peels and decorated with Cu_2_O crystals, displaying a linear response up to 1 mM and a detection limit of 1.2 μM [99]. Moreover, the highly lignocellulosic orange peel waste can also be transformed into innovative acoustic material through ultrasonic treatment [100]. The bioconversion of citrus processing waste to biobased polymers, antimicrobial materials, and adsorbents, is reported in Table 4.

## 6. Solid-State Fermentation of Citrus Processing Waste for Food and Environmental Processing Applications

Solid-state fermentation of citrus processing waste has gained ground for various applications in food and environmental technology (Table 5). A biorefinery approach to valorize orange peels by extracting essential oils, i.e., limonene, and recovering high-activity peroxidase, while bioconverting the remaining cellulose via enzymatic hydrolysis into lactic acid and the bioplastic polyhydroxybutyrate using *Weizmannia coagulans* and *Priestia megaterium*, respectively, was conducted by Mihalyi et al. [101]. Mondal et al. [102] also reported on the enzymatic bioconversion of a mixture of agricultural residues containing orange peels carried out by the fungal strains *Aspergillus niger* SKN1 and *Trametes hirsuta* SKH1 and subsequently valorization of the hydrolysate through fermentation with *Clostridium acetobutylicum* ATCC824 to produce biobutanol. Raw orange bagasse pellets were also subjected to fermentation with a *Clostridium beijerinckii* strain to produce butanol [103]. Citrus processing waste can be utilized by various *Lactobacillus* spp. to achieve lactic acid fermentation. *Lactobacillus casei* 2246 fermented orange peels through a yield of 0.88 g lactic acid/g d.w. under static cultivation [104]. Another example is *L. delbrueckii* subsp. *delbrueckii*, which could utilize the hydrolysate of orange peels to produce D-lactic acid [105]. Solid-state fermentation of orange peels can be achieved by *Aspergillus* spp., e.g., *A. oryzae* and *A. niger* bioconverted orange peels and sugarcane bagasse as well as orange peels to galacturonic acid [106] and citric acid [37], respectively. In addition, cultivation of the alga *Euglena gracilis* on orange peels resulted in β-glucan production [107].

The solid-state fermentation of orange peels and grape pomace by the fungus *Aspergillus awamori* was carried out by Díaz et al. [108] in both packed bed and tray-type bioreactors. They produced a fermentation product with high xylanolytic, cellulolytic and pectinolytic activity that was then applied effectively for orange juice clarification. Moreover, the pectinolytic, cellulolytic and xylanolytic potential of the fungi *Aspergillus niger* BTL, *Fusarium oxysporum* F3, *Neurospora crassa* DSM 1129 and a *Penicillium decumbens* sp. were evaluated under solid-state fermentation of orange peels, revealing that *A. niger* BTL exhibited the highest β-xylosidase, polygalacturonase, invertase and pectate lyase activity, whereas *N. crassa* DSM 1129 demonstrated the highest endoglucanase activity [109]. In addition, Tao et al. [110] performed solid-state fermentation of sweet orange processing waste using a *Eupenicillium javanicum* strain, reporting endoglucanase, β-glucosidase and pectinase activities of approximately 50 U/g and xylanase activity near 105 U/g. Solid-sate fermentation of orange peels using a *Trichoderma viride* strain resulted in high cellulolytic activity, exceeding 400 U/mL [111], while, using *Aspergillus brasiliensis*, led to polygalacturonase activity up to 45 U/g [112]. *Cladosporium* strains were also found to exhibit enhanced endoglucanase, exoglucanase, xylanase, pectinase and amylase activities during both submerged and solid-state fermentation of orange peels [113]. Solid-state fermentation of a mixture of orange peels and exhausted sugar beet cossettes caused the induction of high xylanase and exo-polygalacturonase activities, whereas addition of commercially available cellulases to the fermented material permitted the effective hydrolysis of this food waste [114]. Giese et al. [115] proceeded orange bagasse by the fungal strain *Botryosphaeria rhodina* MAMB-05 to produce a solid-state fermentation product with high pectinase activity. An α-amylase activity of 8.5 U/mL was recorded by Ben Hadj Hmida et al. [116] during treatment of orange peels with *Bacillus cereus*, whereas Ousaadi et al. [117] determined α-amylase activity of 12.19 U/mL by growing a halophilic *Streptomyces* strain in orange peel-derived medium. In addition, Serra et al. [118] achieved the heterologous expression of a polygalacturonase gene from the thermophile *Thermomyces lanuginosus* to the *Komagataella phaffii* yeast, recording recombinant pectinase activity up to 460 U/mL. Moreover, yeasts of *Wickerhamomyces subpelliculosus* could exhibit L-methioninase activity of 94.08 U/mL during orange pulp processing [119].

## 7. Bioactive Compounds and Antioxidant Properties of Raw and Biotreated Citrus Processing Waste

Citrus processing residues can be used as a source for the recovery of bioactive compounds (Table 6) that can be applied as dietary supplements in functional foods [120]. Essential oils, phenolics and pectin as well as cellulosic material can be obtained from orange peels through the implementation of a biorefinery approach [121]. Pulsed electric field of 7 kV/cm, which assists the extraction of bioactive compounds, led to increased recovery of naringin and hesperidin from orange residues, which possess high antioxidant capacity [122], while hesperidin from orange peels could biotransform to the antioxidant diosmetin [123]. Application of a recombinant α-L-rhamnosidase resulted in the hydrolysis of naringin to rhamnose [124], whereas enzymatic hydrolysis combined with ultrasonic treatment permitted the bioconversion of orange processing waste to β-carotene [125].

Apart from the production of hydrolytic enzymes for the food and beverage industry, citrus processing waste through biotreatment can serve as a potential source of recovering high value-added products for food applications, such as antioxidants. Bier et al. [126] evaluated the antioxidant activity of the solid-state fermentation product derived from the bioconversion of limonene present in orange processing waste after inoculation with a *Diaporthe* strain. Sepúlveda et al. [127] valorized the high polyphenolic content of orange processing waste to produce the antioxidant and antibacterial compound ellagic acid at a yield of 19 mg/g of dry orange peel through submerged fermentation. Yu et al. [128] also applied mixed-type fermentation of orange peel pomace using *Lactobacillus casei*, *Aspergillus oryzae* and *Trichoderma koningii* at a microbial species ratio of 7:5:1, resulting in increased antioxidant capacity of the fermented byproduct, with metabolomics analysis indicating the positive impact of biomolecules such as pinoresinol, gentisic acid, quercetin 3-galactoside 7-rhamnoside and quercetin 3-lathyroside. However, a key factor in the development of bioconversion strategies for citrus processing waste into high-value products is the standardization of the bioconversion process.

## 8. Citrus Processing Waste as a Source of Specialized Microbial Starter Cultures

Citrus processing waste is also considered a source for selecting microbial starting cultures. For instance, Zerva et al. [129] reported that spontaneous fermentation of orange processing waste led almost exclusively to the proliferation of lactic acid bacteria and yeasts with potential applications as starting inocula in food and environmental applications. Despite the broad diversity of indigenous microorganisms in citrus processing waste capable of degrading pectin, cellulose and hemicellulose, which are perfectly adapted to citrus residues environment, their application is restricted, and allochthonous microbial strains are often applied during solid-state fermentation [129,130,131]. Therefore, citrus processing waste is considered a valuable source for the isolation of pectin-, cellulose- and hemicellulose-degrading microorganisms or the recovery of specialized mixed cultures consisting of such indigenous microbiota, with potential uses in the food and beverage industry, like in must [132] and juice [130] clarification. Despite the broad diversity of these degrading microbiota in citrus processing waste, the application of indigenous microorganisms capable of degrading pectin, cellulose and hemicellulose, which their growth is perfectly adapted to citrus residues, is restricted, and allochthonous microbial strains are often applied during solid-state fermentation. Indeed, solid-state fermentation of citrus waste with autochthonous microbiota, either in pure or mixed cultures, appears to be advantageous for biotechnological applications in food and environmental technology, and in animal feeding.

## 9. Valorization of Citrus Processing Waste as a Sustainable Ingredient in Animal Feeding

Citrus processing waste can also be valorized in animal feeding by substituting common nutrient compounds in animal diets. For instance, orange processing waste was used as a substitute ingredient in fish diets, such as that of *Labeo rohita*, to replace basic dietary materials like wheat flour and rice bran [133]. Rego et al. [134] evaluated the effects of substituting corn with orange pulp in lamb diets and found that while lamb weight remained unchanged, increasing the proportion of orange pulp in the diet led to reduced fat deposition and muscle area. Researchers have also highlighted orange peels as a promising feed option for small ruminants like dairy ewes [135], which with proper processing, could serve as supplements to improve animal health and nutritional status [136]. Varela et al. [137] reported that rabbit diets with up to 30% orange pulp can be used as a substitute of conventional diets without affecting their digestibility. By using orange pulp as a dietary supplement of laying hens at 7% and 10%, Hussein et al. [138] found that a range of parameters, such as body weight gain, feed intake and feed conversion ratio, as well as egg production, weight and mass, were ameliorated compared to the control. Moreover, the use of orange pulp in the diet of the laying hens increased eggshell weight and thickness and intensified egg yolk color, resulting in a better antioxidant capacity and a more preferable fatty acid composition. However, in a study by Goliomytis et al. [139] where laying hens were dietary supplemented with 9% orange pulp, the improved egg yolk oxidative capacity observed was accompanied with deteriorated performance because of reduced feed intake, which was attributed to the reduced palatability of the citrus pulp. The differences in citrus pulp composition and constituents, such as the percentage of seeds that are rich in tannins and pectin and may affect palatability, may explain discrepancies among published studies. On the other hand, improved meat oxidative stability during refrigerated storage without any adverse effects on the performance of broiler chickens fed with a diet containing 5% citrus pulp [140] suggests that citrus pulp may successfully incorporate in broiler diets. Although citrus processing waste can be used in animal feed as stated, the lack of agronomic standardization may hinder its wider application [141].

## 10. Conclusions

Citrus processing waste is an abundant resource for the recovery of high value-added products, including antioxidants, hydrolytic enzymes and biodegradable packaging materials, and the synthesis of innovative adsorbent nanostructured material. Bioconversion of citrus residues like peels, seeds and pulp, following various biotreatment techniques, such as solid-state fermentation, which is not inhibited by their high content in essential oils, can result in the effective waste management of this agricultural byproduct, reducing environmental impact and resulting simultaneously to the formation of innovative functional compounds within the bioeconomy prism. Bioconverted citrus residues may result in enhanced soil fertility and phytoprotection, whereas citrus processing waste can comprise an alternative feedstuff in animal diets. Such biotreatment approaches can boost agri-food, pharmaceutical and energy sectors in citrus producing countries. Although the employment of the above-mentioned valorization bioengineering methods can boost the economy of local communities in citrus processing countries, issues like the variation in the composition of citrus residues and the type of fermentation method employed should be addressed. Moreover, the legislation framework dealing with the use of agro-industrial residues, including citrus processing waste, and their substrates as functional food and feed additives should be reconsidered and simplified. These biotechnological approaches should also be evaluated through detailed life cycle assessment and advanced techno-economic analyses to assess the feasibility of their implementation. Further research on optimizing the recovery of bioactive compounds and the fermentation process will facilitate the adoption of such sustainable technological solutions.

## Figures and Tables

**Table 1:** Peel content and key biomolecules and bioactive compounds of major citrus peel residues.

	Orange	Lemon	Grapefruit	Pomelo	Tangerine
Peel Portion (% of the whole fruit)	14.3–26.0 [2,3,4]	36.4–48.0 [4,5]	27.8–41.0 [4,6]	46.9–48.6 [4,5]	24.0–55.0 [7,8]
Hesperidin (mg/g dw)	21.8–48.0 [9,10]	6.7–24.5 [11,12]	5.0–7.2 [13,14]	0.7–4.2 [15,16]	21.6–41.3 [12,13]
D-Limonene (% of the EO)	70.0–90.0 [17,18]	48.6–67.1 [19,20]	74.8–75.1 [21,22]	67.6–84.6 [23,24]	71.7–85.1 [25,26]
Pectin (%)	12.5–29.1 [27,28]	15.0–37.9 [29,30]	22.6–27.3 [31,32]	18.8–28.5 [33,34]	12.8–25.6 [35,36]
Cellulose (%)	9.2–11.9 [37,38]	8.2–12.7 [39,40]	27.2 [41]	15.7–21.3 [42,43]	22.5 [44]
Hemicellulose (%)	10.5–14.5 [37,38]	5.3–18.7 [39,40]	6.3 [41]	8.1 [43]	6 [44]
Lignin (%)	0.8–2.2 [37,38]	1.7 [40]	13.1 [41]	0.1 [43]	8.6 [44]
Lipids (%)	1.9 [37]	5 [45]	0.2 [31]	1.6 [43]	1–8.7 [46,47]
Moisture (%)	7 [37]	11.5 [48]	75 [31]	16.1 [43]	6.1 [46]
Ash (%)	3.5 [37]	1.9 [40]	1.5 [31]	3.4 [43]	3.1–4 [46,47]

**Table 2:** Bioenergy recovery from citrus processing waste.

Process	Products	Reference
Anaerobic digestion	Biogas (without pretreatment); Biogas of high methane yield (via D-limonene minimization)	[58,59,60,61]
Dark fermentation	Biohydrogen production	[62,63]
Hydrothermal pretreatment + fermentation	Enhanced biohydrogen production, biobutanol production	[64]
Yeast fermentation	Bioethanol, Lipids for biodiesel production (39% lipid content)	[65,66,67,68]
Lipid extraction from orange seeds	Lipids for biodiesel production (37% content; Fatty Acids: oleic, linoleic and palmitic acids)	[69]
Ensiling	Lactic acid and bioethanol—Enhanced anaerobic digestibility (65% VS, 75% limonene reduction)	[70,71]

**Table 3:** Bioconversion of citrus processing waste to soil fertility enhancers and phytoprotective agents.

Process	Products	Reference
Direct application of CPW	Phytoprotection (against weeds, insects, nematodes, fungi) via essential oils	[73]
Co-composting	Mature compost with low phytotoxicity (mesophilic → thermophilic microbiota)	[74]
Vermicomposting (with *Eisenia fetida*)	Stabilized compost from orange waste	[75]
Compost amendment to soil	Optimal seedling growth (tomato, zucchini) at 7.5% compost rate	[76]
Field application (4 kg/m^2^ orange waste)	Comparable wheat yield to mineral fertilizer	[77]
Application in onion fields	Weed control and doubled onion bulb yield	[78]
Extract of orange juice waste	Bioherbicidal effect (weed germination inhibition/delay without harming crop biomass)	[79]
Use of CPW in pesticide removal	Removal of diazinon and parathion; CPW as biosorbent	[80]
Citrus essential oil	Insecticidal protection against *Callosobruchus maculatus* on chickpeas	[81]
Orange peel essential oils	Control of postharvest anthracnose (*Colletotrichum gloeosporioides*, *C. scovillei*) on mangoes	[82]
CPW as fungal growth substrate	Cultivation of bioprotective fungi (*Beauveria bassiana*, *Trichoderma harzianum*) for biopesticide use	[83]

**Table 4:** Bioconversion of citrus processing waste to produce biobased polymers, antimicrobial materials, and adsorbents.

Process	Products	Reference
Microbial growth on orange peels	PHB (bioplastic) production by *Bacillus cereus* (0.4 g/kg)	[85]
Orange peel extract in biocomposite film	Antimicrobial and antioxidant packaging film (against *Salmonella enterica* and *Escherichia coli*)	[86]
Orange peels in biopolymer composite	Functional packaging: antioxidant and antibacterial activity	[87]
Film with eggshell, pectin and orange EO	Antimicrobial biocomposite (vs *Staphylococcus aureus*, *B. cereus*); good barrier and stress resistance	[89]
Submerged fermentation of CPW	Biosurfactant production by *Bacillus haynesii* E1	[90]
AgNP synthesis using orange peel extract	Antimicrobial silver nanoparticles (active against bacteria and fungi)	[91]
Citrus chitosan-coated selenium nanocomposite	Antifungal activity vs. *Sclerotinia sclerotiorum* (complete inhibition at 0.5 ppm)	[92]
Activated carbon from CPW (TiO_2_-modified)	Arsenic removal (10.9 mg/g, pH 4.2, 3.3 g/L dosage)	[93]
Microwave-activated orange peel carbon	Heavy metal removal: As (V), Se (IV), Cu (II), Cd (II)	[94]
Orange peel as bioadsorbent	Methylene blue dye removal (~112 mg/g capacity)	[95]
Conversion to nanoporous materials	Adsorptive materials for biochemical use	[96]
ZnO-orange-peel porous nanocomposite	Dye removal (>90% for crystal violet and methylene blue)	[97]
Orange peel biochar	DNA adsorption (potential to remove antibiotic resistance genes)	[98]
Electrochemical nitrate sensor (Cu_2_O–carbon)	Pollution monitoring; linear detection to 1 mM, limit: 1.2 μM	[99]
Ultrasonic-treated orange peel	Acoustic insulation material	[100]

**Table 5:** Key fermentation end products of citrus processing waste.

Process	Products	Reference
Biorefinery of orange peels with *Weizmannia coagulans* and *Priestia megaterium*	Limonene, high-activity peroxidase, lactic acid, polyhydroxybutyrate	[101]
Enzymatic bioconversion of orange peel-based agricultural residues using *Aspergillus niger* SKN1 and *Trametes hirsuta* SKH1	Hydrolysate fermented into biobutanol by *Clostridium acetobutylicum*	[102]
Fermentation of orange bagasse pellets by *Clostridium beijerinckii*	Butanol	[103]
Lactic acid fermentation by *Lactobacillus casei* 2246	Lactic acid (0.88 g/g d.w.)	[104]
Fermentation by *L. delbrueckii* subsp. *delbrueckii*	D-lactic acid	[105]
Solid-state fermentation by *Aspergillus oryzae*	Galacturonic acid	[106]
Solid-state fermentation by *Aspergillus niger*	Citric acid	[106]
Cultivation of *Euglena gracilis* on orange peels	β-glucan	[107]
Solid-state fermentation by *Aspergillus awamori*	Hydrolytic enzymes (xylanolytic, cellulolytic and pectinolytic enzymes) for juice clarification	[108]
Solid-state fermentation by *Aspergillus niger* BTL, *Fusarium oxysporum* F3, *Neurospora crassa* DSM 1129, *Penicillium decumbens* sp.	β-xylosidase, polygalacturonase, invertase, pectate lyase, endoglucanase	[109]
Fermentation with *Eupenicillium javanicum*	Endoglucanase, β-glucosidase, pectinase (~50 U/g), xylanase (~105 U/g)	[110]
Fermentation with *Trichoderma viride*	Cellulolytic activity (>400 U/mL)	[111]
Fermentation with *Aspergillus brasiliensis*	Polygalacturonase (up to 45 U/g)	[112]
Fermentation with *Cladosporium* spp.	Endoglucanase, exoglucanase, xylanase, pectinase, amylase	[113]
Fermentation of orange peels + sugar beet cossettes	Xylanase, exo-polygalacturonase; improved hydrolysis with added cellulases	[114]
Fermentation by *Botryosphaeria rhodina* MAMB-05	Pectinase	[115]
Treatment of orange peels with *Bacillus cereus*	α-amylase (8.5 U/mL)	[116]
Fermentation with a halophilic *Streptomyces* sp.	α-amylase (12.19 U/mL)	[117]
Heterologous expression in *Komagataella phaffii*	Recombinant pectinase (460 U/mL)	[118]
Processing by *Wickerhamomyces subpelliculosus*	L-methioninase (94.08 U/mL)	[119]

**Table 6:** Valorization of citrus processing waste to produce bioactive compounds.

Process	Products	Reference
Biorefinery approach on orange peels	Essential oils, phenolics, pectin, cellulosic material	[121]
Pulsed electric field treatment (7 kV/cm)	Increased recovery of naringin and hesperidin	[122]
Biotransformation of hesperidin from orange peels	Antioxidant diosmetin	[123]
Recombinant α-L-rhamnosidase hydrolysis of naringin	Rhamnose	[124]
Enzymatic hydrolysis combined with ultrasonic treatment	Bioconversion of orange waste to β-carotene	[125]
Solid-state fermentation of orange waste by *Diaporthe* sp.	Antioxidant products from bioconversion of limonene	[126]
Submerged fermentation of orange peel waste	Antioxidant and antibacterial ellagic acid (19 mg/g yield)	[127]
Mixed fermentation of orange peel pomace (with microbes)	Increased antioxidant capacity; biomolecules like pinoresinol, gentisic acid and quercetin derivatives	[128]

## Data Availability

No new data were created or analyzed in this study. Data sharing is not applicable to this article.
